# Copy number variation study in Japanese quail associated with stress related traits using whole genome re-sequencing data

**DOI:** 10.1371/journal.pone.0214543

**Published:** 2019-03-28

**Authors:** Bhuwan Khatri, Seong Kang, Stephanie Shouse, Nicholas Anthony, Wayne Kuenzel, Byungwhi C. Kong

**Affiliations:** Department of Poultry Science, Center of Excellence for Poultry Science, University of Arkansas, Fayetteville, AR, United States of America; German Cancer Research Center (DKFZ), GERMANY

## Abstract

Copy number variation (CNV) is a major driving factor for genetic variation and phenotypic diversity in animals. To detect CNVs and understand genetic components underlying stress related traits, we performed whole genome re-sequencing of pooled DNA samples of 20 birds each from High Stress (HS) and Low Stress (LS) Japanese quail lines using Illumina HiSeq 2×150 bp paired end method. Sequencing data were aligned to the quail genome and CNVnator was used to detect CNVs in the aligned data sets. The depth of coverage for the data reached to 41.4x and 42.6x for HS and LS birds, respectively. We identified 262 and 168 CNV regions affecting 1.6 and 1.9% of the reference genome that completely overlapped 454 and 493 unique genes in HS and LS birds, respectively. Ingenuity pathway analysis showed that the CNV genes were significantly enriched to phospholipase C signaling, neuregulin signaling, reelin signaling in neurons, endocrine and nervous development, humoral immune response, and carbohydrate and amino acid metabolisms in HS birds, whereas CNV genes in LS birds were enriched in cell-mediated immune response, and protein and lipid metabolisms. These findings suggest CNV genes identified in HS and LS birds could be candidate markers responsible for stress responses in birds.

## Introduction

Understanding the evolutionary process that leads to divergence in animals requires study of their genetic variation. Genomic variation is a principal factor responsible for phenotypic diversity in animals [1]. Basically, genomic variation can encompass a wide range of alterations from small indels to sometimes deletion or duplication of the entire genome. The deletion or duplication of a certain fragment of DNA causes change in copy number variation (CNV) of genome [2]. CNV is arbitrarily defined as DNA segment that is 1 kb or larger and present at variable copy number in comparison to a reference genome [3]. It is estimated that DNA region that have CNV can account for 4.8–9.5% of human genome and surpass the diversity caused by single nucleotide polymorphisms [4, 5].

Due to their larger sizes and abundances, CNVs are likely to impact functions of many genes and consequently fitness in animals [6]. So far four different mechanisms have been proposed for the formation of CNVs i.e. non-allelic homologous recombination (NAHR), non-homologous end joining (NHEJ), Fork Stalling and Template Switching (FoSTeS) and Retrotransposition [7]. CNVs potentially exert phenotypic diversity in animals through changes in gene structure, gene dosage, and gene expression by exposing recessive alleles [8] or indirectly through the perturbation of regulatory region of genes [9]. Their impacts on individuals can be adaptive or maladaptive in different environmental conditions [10].

Several studies have identified CNVs associated with phenotypic variations and complex disorders in human, such as schizophrenia, developmental delay, mental retardation, autism, systemic lupus erythematosus, diabetes, obesity, psoriasis, neuroblastoma and susceptibility to HIV infection [11–13]. Phenotypic diversity associated with CNV has also been characterized in various domestic animals. The pea-comb phenotype characterized by decrease in comb size in male and female chickens is due to duplication of the first intron of sex determining region Y (SRY)-box5 (*Sox5)* gene [14]. Late feathering phenotype in chicken is due to partial duplication of prolactin receptor (*PRLR)* and sperm flagellar 2 (*SPEF2)* genes [15]. Similarly excessive black pigmentation phenotype in chickens is due to duplication of 130 kb locus containing endothelin 3 (*EDN3)* gene [16]. White coat phenotypes in sheep and pigs are due to duplications of agouti signaling protien (*ASIP)* and KIT proto-oncogene receptor tyrosine kinase (*KIT)* genes respectively [17, 18]. Dorsal hair ridge in Rhodesian and Thai dogs and their susceptibility to dermoid sinus is caused by duplication of fibroblast growth factors (*FGF3*, *FGF4*, and *FGF19)* and oral cancer overexpressed 1 (*ORAOV1)* genes [19]. CNVs have also been reported to be associated with disease resistance and developmental disorders in animals. Loss of MHC class I antigen-presenting proteins are associated with Marek’s disease resistance in chicken [20]. Gain of class II major histocompatibility complex transactivator (*CIITA)*, a trans-activator of MHC II is associated with nematode resistance in cattle [21]. Likewise, cone-rod dystrophy 3 [22], startle diseases in dogs [23], and osteopetrosis, abortion and stillbirths in cattle have been linked to CNV [24, 25]. From these findings we hypothesize that CNVs can be important biomarkers for phenotypic traits or disease resistance in animals.

In this study we have performed CNV analysis in whole genome re-sequenced data of high and low stress lines of Japanese quail with a specific focus to identify full length genes within CNV regions (CNVRs). These genes could be relevant for divergence and adaptation of the two lines of quail. Two genetically distinct line of Japanese quail named as high stress (HS) and low stress (LS) were selected for divergent plasma corticosterone response to restraint stress in the 1980s [26]. Since then these two lines have been used as stress responding animal models in poultry. In LS line, the mean corticosterone level is approximately one-third lower compared to HS line. As compared to HS line, LS line is less fearful and more social. It has higher body weight and egg production ability, and reduced heterophil/lymphocyte ratio. It shows lower stress-induced osteoporosis, accelerated onset of puberty, and heightened male sexual activity and efficiency compared to HS line [26, 27].

Currently four basic strategies such as read pair, read-depth, split-read and sequence assembly are being used for CNV detection in next generation sequencing data. We used the software tool CNVnator [28] that works under read-depth approach as the most suitable method to detect CNVs in our data and address our hypothesis. CNVnator is suggested to have many advantages over other methods with respect to accurate CNV detection, precise break point resolution, and detection of different sizes of CNVs, from a few hundred bases to several megabases in the whole genome. In addition, CNVnator has high sensitivity (86–96%), low false discovery rate (3–20%) and high genotyping accuracy (93–95%) [28, 29]. In this this study we have detected major differences in CNV among genes that might potentially contribute to genetic differences and phenotypic divergence in HS and LS lines of Japanese quail.

## Materials and methods

### Ethics statement

This study was conducted following the recommended guidelines for the care and use of laboratory animals for the National Institutes of Health. All procedures for animal care were performed according to the animal use protocols that were reviewed and approved by the University of Arkansas Institutional Animal Care and Use Committee (IACUC Protocol #14012).

### Birds and DNA sequencing

The early process of development and selection of HS and LS lines of Japanese quail for their plasma corticosterone response to immobilization for up to 12 generations was explained by Satterlee and Johnson (1988) [26]. Since then, an independent random mating condition has been used for their maintenance [30–32]. These research lines were shipped to University of Arkansas at generation 44 from Louisiana State University and maintained at Arkansas Agricultural Experimentation Station, Fayetteville, AR [27].

We used adult male HS and LS birds for this study because of their stable physiology. We collected blood samples (3ml) from 20 birds each from HS and LS lines. Genomic DNA was purified from each sample using QiaAmp DNA mini kit (Qiagen, Hilden, Germany) following manufacturer’s method. DNA quality was assessed using NanoDrop 1000 (Thermo Scientific, Waltham, MA) and agarose gel electrophoresis. Twelve samples showing highest quality per line were pooled to represent each line. Library preparation and Illumina sequencing for the pooled DNA samples were performed by the Research Technology Support Facility at Michigan State University (East Lansing, MI) using Illumina HiSeq 2×150 bp paired end read technology.

### Data quality assessment and sequence assembly

We used the FastQC tool (v0.11.6) to assess the quality of raw reads obtained after sequencing in form of FASTQ files (http://www.bioinformatics.babraham.ac.uk/projects/fastqc/). After quality assessment, the low quality reads were trimmed out using Trimmomatic tool (v0.32) [33]. The clean reads were then mapped onto the Japanese quail reference genome obtained from NCBI (https://www.ncbi.nlm.nih.gov/genome/113) using Bowtie2 (v2.3.3.1) with the default settings for the parameters [34]. We removed PCR duplicates using rmdup command line of SAMtools (v0.1.19) and SAMtools was further used to convert SAM to BAM files and then to sorted BAM files to save run time in subsequent analysis [35].

### CNV detection and copy number estimation

We used CNVnator software (v0.3.3) to predict CNV in sorted BAM files relative to reference quail genome [28]. Optimal bin sizes of 1200 and 1500 were chosen for HS and LS respectively according to author’s recommendations, in which the ratio of average read-depth signal to its standard deviation was between 4 and 5 [1, 28, 36]. All the CNV calls in both HS and LS samples were greater than 1 kb. CNV calls were filtered according to criteria recommended by Abyzov et al. [28] CNV showing P-value <0.01 (e-val1 calculated using t-test statistics), size >1 kb, and q0 < 0.5 (q0: fraction of mapped reads with zero quality) were filtered and used for downstream analysis.

We estimated gene copy number (CN) in HS and LS birds across genome length using the “-genotype” option of CNVnator. We wrote a custom bash script and retrieved CNV genes from CNVRs of HS and LS lines using RefSeq genes from NCBI and BEDOPS tool (v2.4.30) [37].

### Real time quantitative PCR validation of CN

Real time quantitative PCR (qPCR) was used to validate CNVs detected by CNVnator in 16 birds each from HS and LS lines. A total of 9 genes showing CNV were randomly chosen and primers were designed using Primer3 software and listed in Table 1.

**Table 1:** Primers used for validation of CNV by qPCR. β-actin was used as internal control for qPCR.

Gene	Forward	Reverse	size
NPTN	TGTCTGCACTGCCTATCAAG	ACGTTGTGTTTCCCATGGTA	158 bp
UBA7	TTGAACTCATCACGAGCCCA	TTTGGTGTCCCATCCCATCT	140 bp
RPHA	AACAGCAGGAAGCTGGGAAT	TCTGCAGGTGCAGCAATGCT	140 bp
CACNG2	TAGAGGAGGATCCACTCAGA	ACAGGATGTGCCAGACCTGA	140 bp
LRRC16B	TCTGCTTGGGATTCCACTGA	AGACTGGGCAACCATCTCTA	160 bp
PCF11	ACAGACCTCTTCCAGTCTAG	ATACATCCACCACTGCCCTT	124 bp
CBFA2T2	AGAGGATATCTGCTGGTAAC	GAGCACGTACTTCAGGTAGA	142 bp
PIH1D3	TGCTGCTGTGACGTGGAATT	GAGACTTGCCAACGTTCTGA	140 bp
FAM219A	ACAGCAGAGATACAGCAGAG	TTGTTGGAGCCCTGCTATTA	140 bp
β-Actin	CTCCTCCTCCCACCCATTTC	GCAGGGACTTCCTTTGTCCC	121 bp

Primers specificities were checked using Primer-BLAST tool of NCBI. A segment of the β-actin gene, which is present in two copies per diploid and showed no CNV in either line of quail, was chosen as control in all reactions. Five nanogram of genomic DNA was subjected to qPCR (total volume of 25 μL) in triplicate reactions using ABI prism 7500HT system (ThermoFisher Scientific) with PowerUp SYBER Green Master Mix (ThermoFisher Scientific). The conditions of real-time qPCR amplification were as follows: 1 cycle at 95°C (10 min), 40 cycles at 95°C (15 s each), followed by 60°C for 1 min. We used ΔΔCt method for calculating relative copy number of each gene. First, the cycle threshold (Ct) value of each gene was normalized against the control gene, and then ΔCt value was determined between test gene and reference gene predicted as normal copy number by CNVnator. Finally values around 3 or above were considered as duplications or gain and around 1 or less as deletion or loss.

### Functional annotation of CNV genes and network identification

We analyzed genes retrieved from CNVRs in terms of gene ontology and molecular networks using Ingenuity Pathway Analysis (IPA; http://www.ingenuity.com). We imported lists of unique genes identified in CNVRs of HS and LS lines of quail into IPA separately and subsequently mapped to their corresponding annotations in the Ingenuity Pathway Knowledge Base. IPA identifies networks accommodating these unique genes in comparison with comprehensive global network. IPA illustrates each molecular network with an assigned relevance score, the number of focus molecules, and top functions of the network. During analysis, we set each network to the limit of 35 molecules by default and only human was chosen for the species option. We used experimentally observed evidence for the confidence level. Finally the identified networks were presented as network graphs that show biological relationship among molecules. Molecules in network graphs are represented by nodes, distinguished by their shapes based on their functional category, and are connected by distinct edges based on interaction among molecules.

## Results and discussion

### Genome re-sequencing and distribution of CNVs

We performed whole genome resequencing of pooled DNA samples from 12 birds each from HS and LS lines of the quail and produced ~250 and ~257 million reads of 150 bp respectively. Of those, ~85 and ~84 million reads were mapped to the reference genome (NCBI/*Coturnix japonicia*) and their respective depth of coverage reached to ~41x and ~42x for HS and LS (Table 2).

**Table 2:** Sequencing and mapping data of high and low stress lines of Japanese quail.

Line	# of raw reads	# of mapped reads	Coverage
HS	250,617,546	85,577,152	41.45x
LS	257,535,422	84,195,797	42.59x

We used CNVnator tool to call CNVs from the mapped data and considered calls (deletions or duplications) ≥1 kb length in our analysis which makes more reliable to detect CNVs when used CNVnator [28]. We chose CNVnator tool because it works based on read-depth approach with a concept that the depth of coverage of a genome is positively correlated with copy number of that region [38]. Furthermore, CNVnator can detect large CNVRs with maximum sensitivity even at low coverage, and the reliability of a CNV call actually increases with the size of event [28, 38, 39].

A total of 262 and 168 CNVRs were identified in HS and LS lines, respectively. Among these, 235 were deleted and 27 duplicated CNVRs in HS, and 148 deleted and 20 duplicated CNVRs in LS lines (Table 3).

**Table 3:** Summary of CNV in high and low stress lines of Japanese quail.

Line	CNVnator bin size	Average RD per bin ± StDev	# of CNVRs	# of deletions	# of duplications	Deletion (Mb)	Duplication (Mb)	Total CNV (Mb)	Average CNV size (Mb)
HS	1200	78.1745± 17.0184	262	235	27	13.80	1.32	15.20	0.05
LS	1500	55.9714 ± 13.2859	168	148	20	17.02	1.15	18.17	1.08

**Fig 1:**
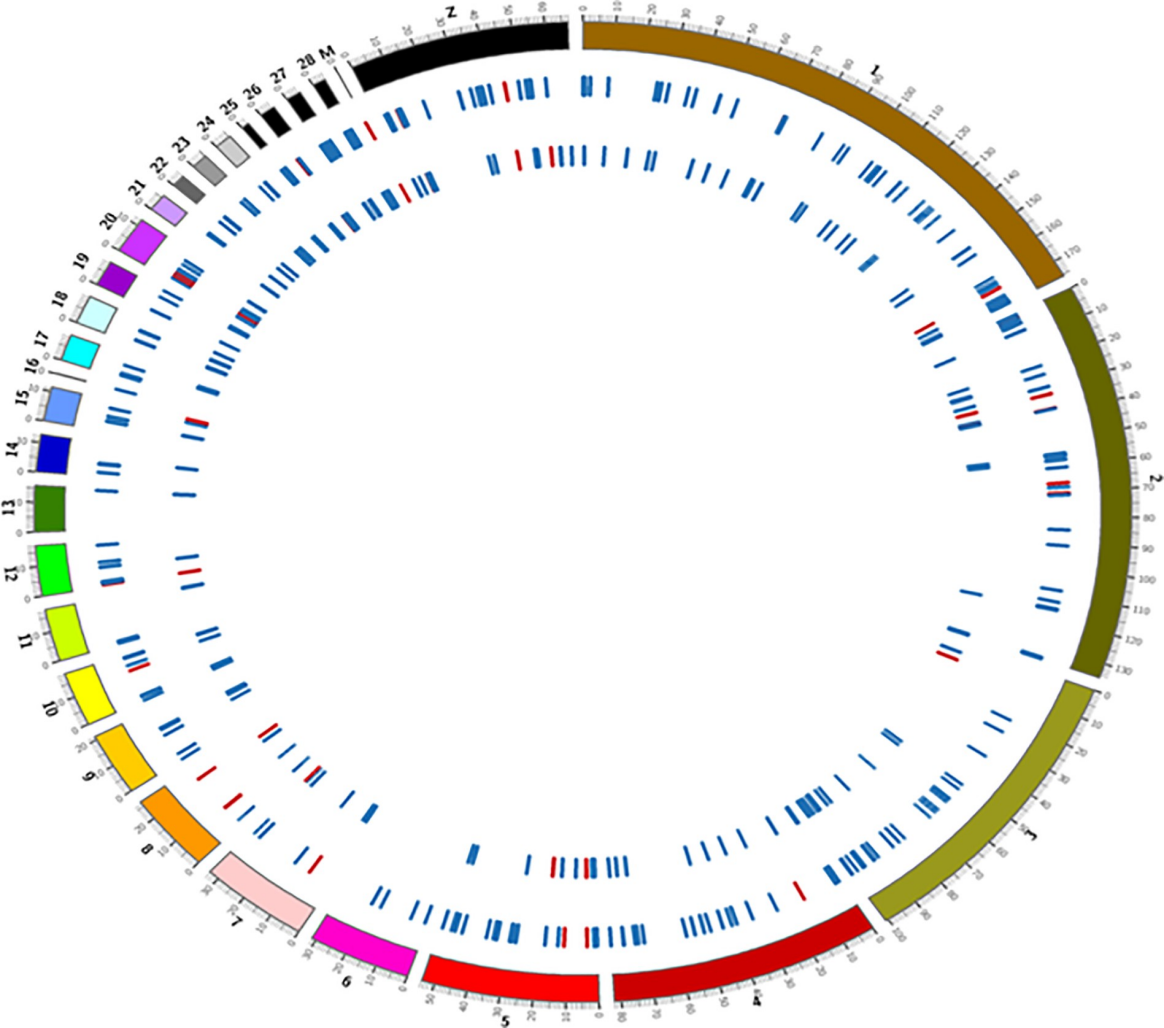
Genome-wide distribution of CNVRs in quail. CNVRs are represented in individual tracks as bars, where the outer track depicts CNVRs in HS and inner in LS line of quail. In the tracks, CNVRs indicated by blue bars are deletions and red bar are duplications with respect to the reference assembly.

The distribution of deletions and duplications over different chromosomes of the quail genome is shown in Fig 1. Interestingly, there were no CNVRs in chromosome 6 and 16 of LS line but were present in HS line. The number of CNVRs in each chromosome was proportional to its length. Replication and recombination based mechanisms have been suggested as possible events for the CNVs formation across genome of an organism. The recombination rate is higher in longer DNA; therefore it can be the reason for more CNVRs present in large chromosomes in our study [8, 40]. The chromosome 16 in chickens has the major histocompatibility complex (MHC) genes that encode key proteins regulating aspects of immune response [41]. A study by Huff et al. reported HS birds more susceptible to *Salmonella* species as compared to LS birds [27]. Therefore, the deletion event detected in chromosome of 16 of HS birds might be the cause for more susceptibility of HS birds to diseases.

**Fig 2:**
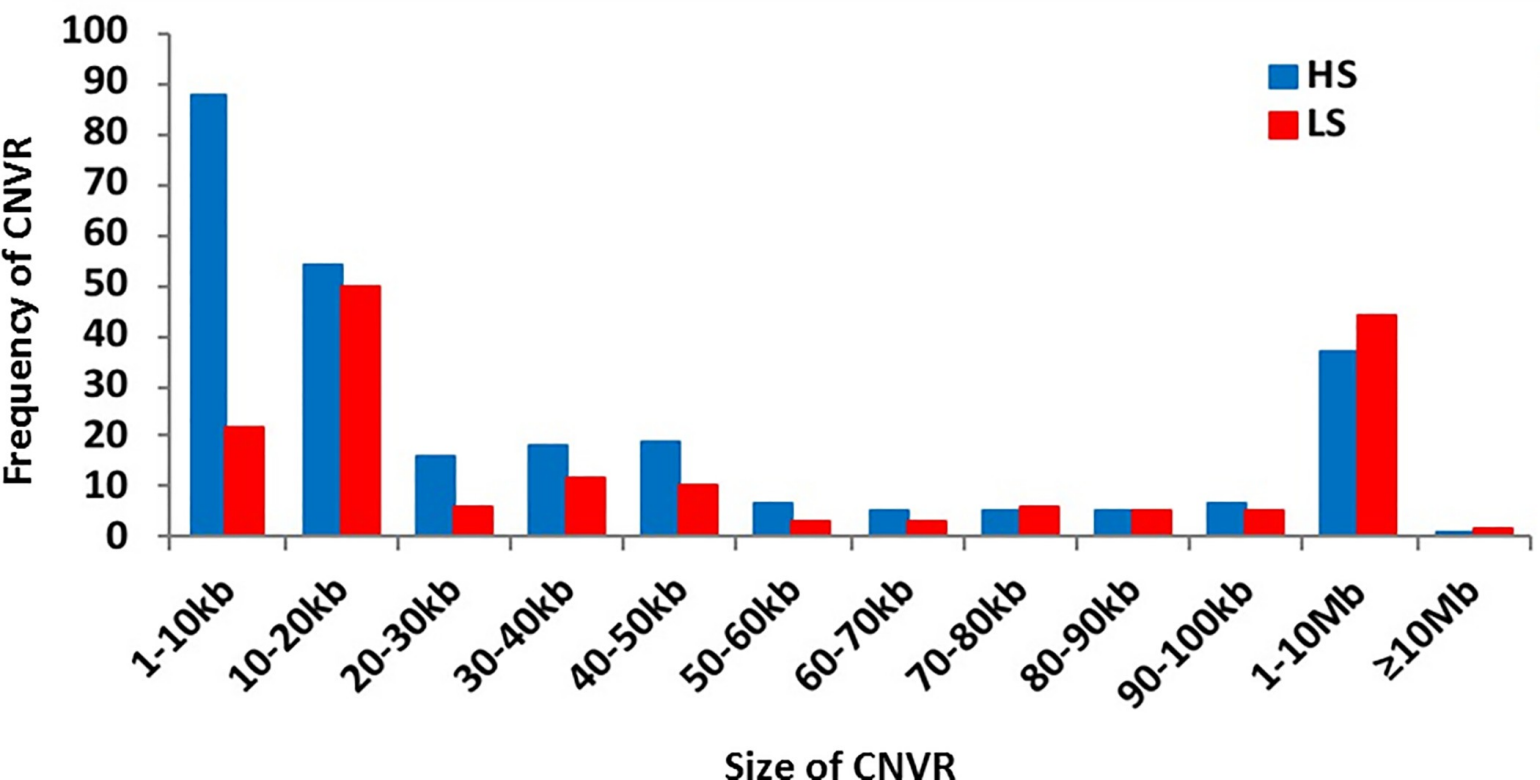
Size and frequency distribution of CNVRs in HS and LS lines of quail.

We found fewer copy numbers with zero state deletions i.e. the genes are completely deleted, compared to one state deletion in both HS and LS lines of quail (S1 Table), which was similar to that observed in chickens [42]. The deletions outnumber duplications by a ratio of 8.70:1 in HS and 7.4:1 in LS (Table 3), which is consistent with previous studies where more deletion events were discovered as compared to duplications [43]. The length of CNVRs ranged from 6.0–1341.6 kb in HS and 7.5–1101 kb in LS lines (Fig 2). The total length of deleted CNVRs accounted for 13.8 Mb in HS and 17.02 Mb in LS lines. Similarly, the total lengths of duplicated CNVRs were 1.32 Mb in HS and 1.15 Mb in LS lines. The average lengths of CNVRs were ~50 kb in HS and ~100 kb in LS lines (Table 3). The CNVRs covered 1.6 and 1.9% of quail genome in HS and LS, respectively. We found the amount of quail genome affected by CNVs similar to that reported for chickens (1.42%, 2.61%) [40, 44], dogs (1.08%) [45], and Holstein cattle (1.61%) [46] but lower than in swine (4.23%) [47], mice (6.87% or 8.15%) [42] and human (5.9%, 12%) [2, 48]. However, these values could be affected by sample size, diversity of samples, sequencing technology and CNV calling methods used in the studies [42].

### CNV validation using qPCR

In this study, we used pooled DNA samples from each line for whole genome re-sequencing (Explained in methods above). However, we validated individual genes associated with CNV in 16 birds each from HS and LS lines using qPCR. We randomly selected 9 different genes each from 9 different CNVRs predicted by CNVnator for their validation. We used ΔΔCt method for determining relative CN of the genes. We found ~80% of our qPCR results agreed with the CN state predicted by CNVnator (Table 4).

**Table 4:** Experimental validation of 9 CN genes using qPCR in larger number of HS (16) and LS (16) birds.

CNV Type	Coordinates	Gene	Copy Number (CNVnator)	Copy Number (qPCR)
Deletion	chr10:1573201–2205600	NPTN	1.53	1.27
Deletion	chr12:1348801–1467600	UBA7	1.42	0.72
Deletion	chr15:5568001–5653200	RPHA	1.38	1.19
Deletion	chr1:47198401–47250000	CACNG2	1.35	1.39
Deletion	chr1:6001–28800	LRRC16B	1.00	1.43
Duplication	chr1:169119601–169160400	PCF11	33.60	1.33*
Duplication	chr20:1994401–2090400	CBFA2T2	25.90	20.57
Deletion	chr4:1858801–2012400	PIH1D3	1.45	1.46
Duplication	chrZ:7086001–7182000	FAM219A	7.80	1.03*

*Indicate inconsistency between CNVnator output and qPCR result

The result clearly showed that there is difference in CNV in 9 genes between HS and LS lines of quail. Thus, difference in CNV observed in genes in between HS and LS lines can be the reason for their phenotypic variations.

### Gene content of CNVRs and bioinformatics analysis

We wrote a custom bash script, used BEDOPS tool and reference genome annotation file (in GFF format) of Japanese quail from NCBI to extract genes from CNVRs of both the lines. We retrieved a total of 948 genes overlapped within CNVRs in HS and 982 in LS lines. The total number of deleted genes was 895 in HS and 922 in LS lines and the number of their respective duplicated genes was 53 and 60. Among the deleted genes, 436 were uniquely deleted in HS and 471 in LS lines (Table 5).

**Table 5:** Number of genes associated with CNVRs in high and low stress lines of Japanese quail.

Quail Lines	Total # of CNV Genes	# of Deleted Genes	# of Duplicated Genes	# of Unique Deleted Genes	# of Unique Duplicated Genes
HS	948	895	53	436	18
LS	982	922	60	471	22

Similarly, we found 18 uniquely duplicated genes in HS and 22 in LS lines (Table 5). Lists of uniquely deleted and duplicated genes in HS and LS lines are shown in S2 Table. Structural genetic variations have been known to accumulate during inbreeding process in animals [49]. However, effect of the inbreeding process in accumulation of genetic variation in quail populations was not known to date. We have identified several hundred genes that were fully deleted in HS and LS lines of quail, which supports a phenomenon of perpetual gene turnover in the two quail populations and their genetic differences. Duplication of whole genes has been known to impact gene expression by altering gene dosage [50, 51]. If a duplication of a gene is adaptive, it is usually favored and retained more frequently in a population [1]. We found 23 genes in HS and 32 in LS lines that have, on average, 10 or more copies and are considered as high copy number genes. These gene lists included both annotated and unannotated genes with 11 genes having compatible copy number between HS and LS lines (S3 Table). The high copy number annotated genes in HS included PCR11 cleavage and polyadenhylation factor (PCF11), ankyrin repeat domain 42 (ANKRD42), obscurin, cytoskeletal calmodulin and titin-interacting RhoGEF (OBSCN), chromosome 2 H6orf52 homolog (C2H6orf52), nucleoporin 153 (NUP153), core-binding factor alpha subunit 2 (CBFA2T2), syntrophin alpha 1 (SNTA1), and dynein axonemal intermediate chain 1 (DNAI1) and in LS lines were PCF11, ANKRD42, and hydroxysteroid dehydrogenase like 2 (HSDL2). The high copy number genes were associated with cellular assembly and organization, cellular morphology, nervous system development and function (S4 Table).

We used IPA to characterize the biological functions, describe molecular interaction networks and canonical pathways implicated by uniquely deleted genes in HS and LS lines. We identified five canonical pathways significantly (p-value < 0.01) enriched by deleted genes in HS lines (Table 6).

**Table 6:** Uniquely deleted genes in HS and LS lines of Japanese quail associated with canonical pathways.

Canonical Pathways	Molecules
***HS line*:**	
Phospholipase C Signaling	ARHGEF11, ARKGEF12, BTK, HDAC5, ITGA3, ITPR1, MEF2B, MEF2D, MPRIP, PLA2G3, PLD6
Neuregulin Signaling	CDK5R1, ERBB2, GRB7, ITGA3, PIK3R2
Reelin Signaling in Neurons	ARHGEF11, ARKGEF12, CDK5R1, ITGA3, MAPT, PIK3R2
ERK Signaling	MAP2K5, MEF2B, MEF2D, NTRK1, SH2D2A
CD27 Signaling in Lymphocytes	CASP9, MAP2K5, MAP3K13, MAP3K14
***LS line*:**	
Type II Diabetes Mellitus Signaling	ACSBG2, ADIPOR2, CACNA1G, CACNA2D4, CACNG3, PIK3C2B, PRKCB, SLC27A3
GP6 Signaling Pathway	COL16A1, COL18A1, COL5A1, COL6A1, COL6A2, COL9A2, PIK3C2B, PRKCB
nNOS Signaling in Skeletal Muslce cells	CACNA1G, CACNA2D4, CACNG3, NOS1
Hepatic Fibrosis / Hepatic Stellate Cell Activation	COL16A1, COL18A1, COL5A1, COL6A1, COL6A2, COL9A2, ECE1, SMAD7
Role in CHK Proteins in Cell Cycle Checkpoint Control	CDKN1A, RAD9A, RFC5, SLC19A1

The pathways include: Phospholipase C Signaling, Reelin Signaling in Neurons, ERK5 Signaling, CD27 Signaling in Lymphocytes and Neuregulin Signaling. Similarly six canonical pathways were significantly (p-value <0.01) enriched by deleted genes in LS lines which includes: Type II Diabetes Mellitus Signaling, GP6 Signaling, nNOS Signaling in Skeletal Muscle Cells, Hepatic Fibrosis/Hepatic Stellate Cell Activation, and role of CHK Proteins in Cell Cycle Checkpoint Control (Table 6). We found the top diseases and bio functions of the deleted genes in HS line related to endocrine system disorders, organismal injury and abnormalities, neurological disease, and gastrointestinal disease. Similarly, the top diseases and bio functions of deleted genes in LS line were related to endocrine system disorder, organismal injury and abnormalities, connective tissue disorders, and reproductive system disease (Table 7).

**Table 7:** Uniquely deleted genes in HS and LS lines of Japanese quail associated with top disease and bio functions.

Name	p-value	# of Molecules
***HS line*:**		
Neurological Disease	1.46E-02–2.11E-04	21
Endocrine System Disorder	1.46E-02–2.84E-05	7
Organismal injury and Abnormalities	1.46E-02–2.84E-05	76
Gastrointestinal Disease	1.46E-02–2.84E-05	12
***LS line*:**		
Endocrine System Disorder	2.80E-02–5.63E-05	14
Organismal Injury and Abnormalities	2.80E-02–1.66E-05	130
Connective Tissue Disorder	2.80E-02–1.66E-05	14
Reproductive System Disease	2.09E-02–1.66E-05	32

The uniquely deleted genes in HS involved in endocrine system disorder are listed in Table 8.

**Table 8:** Uniquely deleted genes in HS and LS lines of Japanese quail associated with endocrine system disorder.

***HS line*:**			
**Symbol**	**Entrez Gene Name**	**Location**	**Type(s)**
CDK5R1	cyclin dependent kinase 5 regulatory subunit 1	Nucleus	kinase
CSF3R	colony stimulating factor 3 receptor	Plasma Membrane	transmembrane receptor
ERBB2	erb-b2 receptor tyrosine kinase 2	Plasma Membrane	kinase
HSD11B2	hydroxysteroid 11-beta dehydrogenase 2	Cytoplasm	enzyme
POLE	DNA polymerase epsilon, catalytic subunit	Nucleus	enzyme
POLE3	DNA polymerase epsilon 3, accessory subunit	Nucleus	enzyme
TRIM29	tripartite motif containing 29	Cytoplasm	transcription regulator
***LS line*:**			
AMH	anti-Mullerian hormone	Extracellular Space	growth factor
CACNA2D4	calcium voltage-gated channel auxiliary subunit alpha2delta 4	Plasma Membrane	ion channel
CDKN1A	cyclin dependent kinase inhibitor 1A	Nucleus	kinase
COL16A1	collagen type XVI alpha 1 chain	Extracellular Space	other
COL18A1	collagen type XVIII alpha 1 chain	Extracellular Space	other
COL5A1	collagen type V alpha 1 chain	Extracellular Space	other
COL6A1	collagen type VI alpha 1 chain	Extracellular Space	other
COL6A2	collagen type VI alpha 2 chain	Extracellular Space	other

Also, LS line has a network associated with lipid metabolism in deletion. Thus, in contrast to LS line, canonical signaling pathways in HS are related to regulation of immune response, stress and neurological diseases. Therefore, a higher level of mean corticosterone level seen in HS compared to LS lines may be associated with the genes with CNVs. These differences might implicate CNV as an adaptive change in response to restraint stress between HS and LS lines of Japanese quail. This type of adaptive variation at DNA level can improve the fitness of organisms to new and challenging environments [52].

We identified a total of 17 gene networks in HS and 18 in LS lines with score not less than 10 (S4 Table) among which 4 different networks in HS and 5 in LS lines were significant interaction networks involved in nervous and endocrine systems development (Table 9).

**Table 9:** Significant interaction networks of uniquely deleted genes involved in nervous system and endocrine development in HS and LS lines of quail.

***HS line*:**				
**SN**	**Molecules in Network**	**Score**	**Focus Molecules**	**Top Diseases and Functions**
1	14-3-3, APH1A, ATP6V0D1, ATP6V1A, ATP6V1G1, atypical protein kinase C, BSN, CAMK2N2, CaMKII, ERK1/2, Glycogen synthase, GPATCH8, Growth hormone, IBA57,IL1RAPL1,INSRR,LLGL1, LSG1, MIOX, MLXIPL, NECTIN1, PEBP4, PP1 protein complex group, PPP1R9B, Proinsulin, pyruvate kinase, RAB3A, RASD1, RPH3A, Secretase gamma, STX1A, STXBP1, TNFRSF13B, Vacuolar H+ ATPase, VWA5B2	37	24	Cell-To-Cell Signaling and Interaction, Cellular Assembly and Organization, Nervous System Development and Function
2	AGMAT, AMPK, BHLHE40, CDC25A, Cg, Ck2, Creb, DUSP23, FAM3D, FSH, GABPB2, Gsk3, HDL, Lh, NCAN, NCL, Nr1h, NUDT15, NUP153, OSBPL2, p70 S6k, PDGF BB, PEPCK, phosphatase, PI3K (family), Pkc(s), POLE, POLE3, PRUNE1, RNA polymerase II, Rnr, SLC36A4, SREBF1, SUGP1, UBTF	24	18	Cancer, Endocrine System Disorders, Gastrointestinal Disease
3	1700030F18Rik, AKNA, APP, ARMC9, ATAT1, ATXN7L3, C16orf78, C4orf46, CARMIL3, CBFA2T2, CD40, CSAG1, DBF4B, DNAJB7, EPB41L4A, FAM212A, GLRA4, GPR6, GPR12, GPR15, GPR61, GPR78, GPR85, JTB, LMF2, MARCH10, MED9, NUP62CL, OCEL1, RXFP3, SLC13A3, SPEN, SRPK2, TMEM41A, VIPR2	17	14	Cell-To-Cell Signaling and Interaction, Inflammatory Response, Nervous System Development and Function
4	ADH7, B3GNT7, BAG6, CADM3, CTRC, Epsin, ESR2, FAM84B, FGD2,HEBP1, KAZALD1, KLHL12, LSM12, Macf1, MRPL55, NAA38, NBPF10 (includes others), OTP, PABPC5, PCMTD2, POU5F1, RALBP1, Rplp1 (includes others), RUNDC3A, SDK1, SLC5A7, SLC6A1, SMAD4, SNRNP25, TBRG1, TCTA, Ubb, UBC, UBL7, ZFHX3	11	10	Nervous System Development and Function, Neurological Disease, Organ Morphology
***LS line*:**				
**SN**	**Molecules in Network**	**Score**	**Focus Molecules**	**Top Diseases and Functions**
1	ACSBG2, ADAMTS9, ADAMTS15, ARHGEF9, ASB18, ATRN, CACFD1, COPS5, CREB3, GCFC2, HSD11B1L, LSM12, MGST2, NUDT1, PGAM5, PHRF1, PPP1CA, PPP1R15B, PRPF6, PRPF39, RELL2, RHEB, RIMS3, RNPC3, RRP7A, SF3A2, SNRNP35, SNRPE, TFIP11, TMEM222, U4 snRNP, U5 snRNP, U6 snRNP, VCAN, ZMAT5	19	15	Developmental Disorder, Hereditary Disorder, Neurological Disease
2	20s proteasome, 26s Proteasome, Alpha tubulin, AMPK, Calcineurin protein(s), CDT1, CPEB1, Cyclin A, Cyclin D, cytochrome C, cytochrome-c oxidase, DFFB, EIF4G3, ELP3, ERK, HISTONE, Histone H1, MEAF6, Mitochondrial complex 1, MRPL48, MTORC2, NFE2L1, Nos, NOS1, OAZ1, PARP, PCDH1, PDE3A, PP2A, PPME1, Ppp2c, PRKAA, Rb, SURF1, TIP60	17	14	Hereditary Disorder, Metabolic Disease, Neurological Disease
3	AKT1, AMIGO2, ARHGAP33, C1orf174, CCL5, CIART, CREB1, CSRNP1, ETNK2, GPR65, GPR83, IGSF9B, JPT1, MMP14, MMP23B, NGF, NR3C1, NRBP2, NTSR1, P2RX3, PCOLCE, RAP1GAP2, SLC17A6, SORCS3, SPATA20, SPOCK3, SRPK2, SRXN1, STON1, TIPARP, VPS26B, VSTM2L, YPEL4, ZDHHC5, ZNF395	11	10	Cell Morphology, Cellular Function and Maintenance, Nervous System Development and Function

**Fig 3:**
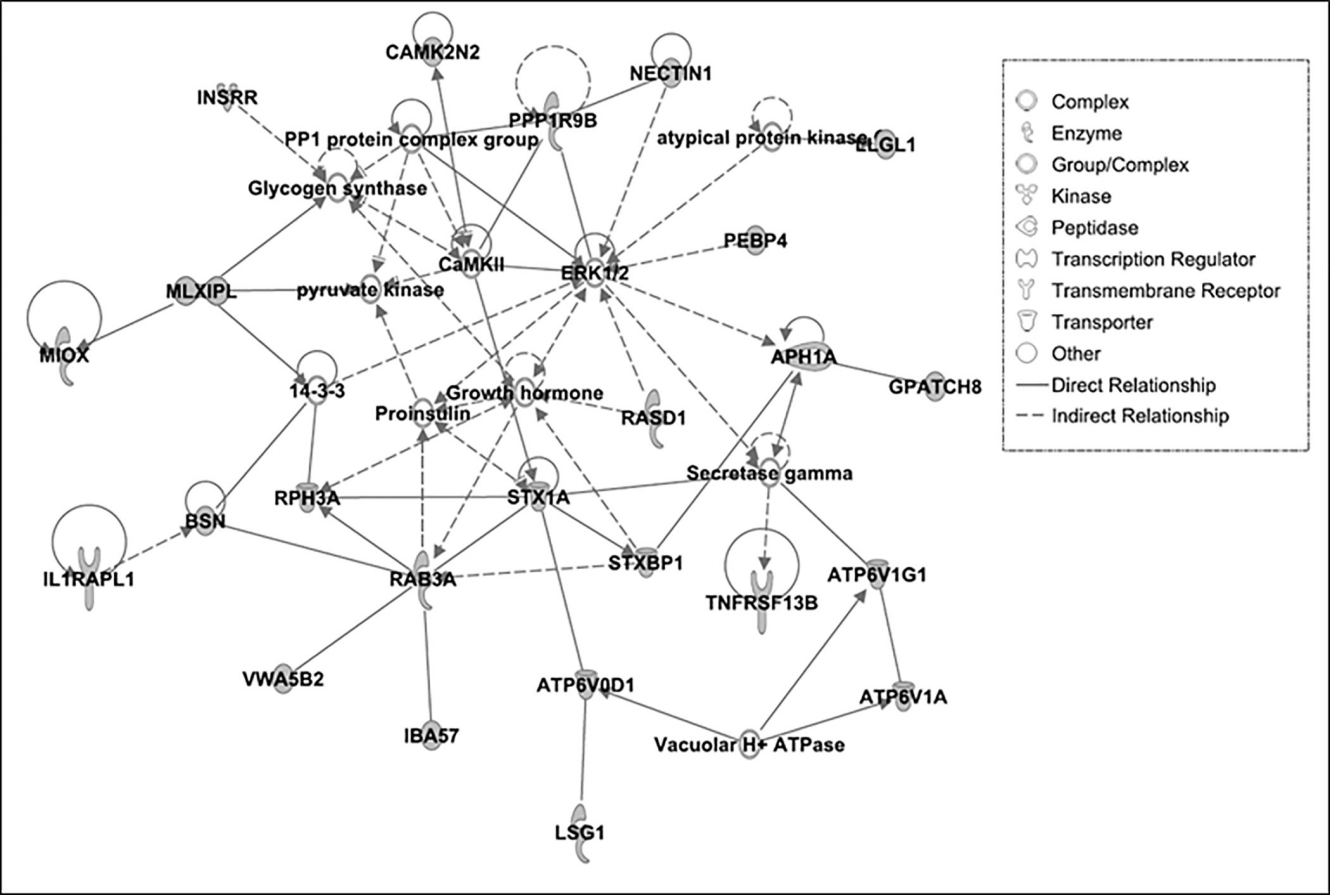
Top-scoring multi-gene network associated with Cell-To-Cell Signaling and Interaction, Cellular Assembly and Organization, Nervous System Development and Function in HS line of quail. The deleted genes are molecules in gray.

Here a score of 10 implies that there will be less than a 10^−10^ probability that the genes in the network are associated with each other by chance. The topmost network involving deleted genes in HS line was specifically associated with cell to cell signaling and interaction, cellular assembly and organization, nervous system development and function (Fig 3). The genes associated with loss in this network are involved in signaling pathways of ERK1/2 connected to CaMKII (Ca^2+^/calmodulin-dependent protein kinase II), PPP1R9B (Protein phosphatase 1 regulatory subunit 9B), APH1A (Aph-1 homolog A), proinsulin and growth hormone. CaMKII, PPP1R96, and APH1A are involved in nervous system development and functions. CaMKII functions in various cells by phosphorylating proteins involved in synaptic plasticity, electrical excitability and neurotransmitter synthesis [53]. PPP1R9B gene is expressed in dendritic spines and plays a role in receiving signals form the central nervous system [54]. APH1A gene encodes a component of gamma secretase complex that is involved in proteolysis of amyloid precursor protein [55]. The connection in this network therefore suggests loss of CaMKII, PPP1R9B, APH1A and other genes in this pathway may impair functional interactions of ERK1/2 signaling pathway with growth hormones, proinsulin and secretase gamma. This may be a reason for reduced growth rate and low basal weight observed in HS compared to LS birds.

**Fig 4:**
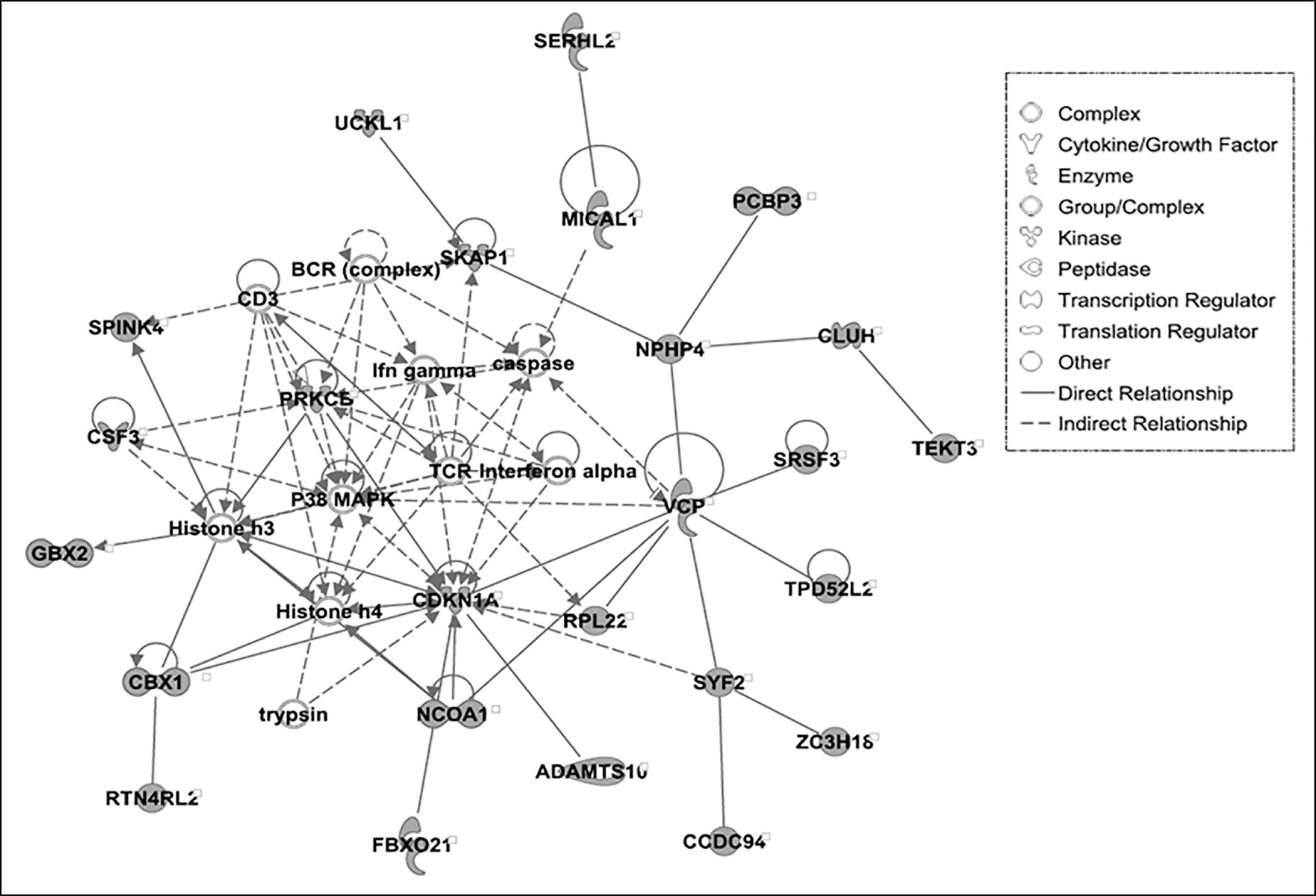
Top-scoring multi-gene network associated with Cell-mediated Immune Response, Cellular Development, Cellular Function and Maintenance in LS line of quail. The deleted genes are molecules in gray.

Reduced heterophil/lymphocyte ratio is observed in LS compared to HS line of Japanese quail [27]. Interestingly, we found humoral immune response in HS and cell-mediated immune response in LS lines associated with deleted genes (S4 Table). The gene network associated with cell-mediated response in LS lines is shown in Fig 4. In this network, the deleted genes are associated with signaling pathway of P38 MAPK connected to CDKN1A (cyclin dependent kinase inhibitor 1A), PRKCE (protein kinase C epsilon) and CSF3 (colony stimulating factor 3). The protein encoded by CDKN1A inhibits cyclin-cyclin-dependent kinase2 and function in regulation of cell cycle progression at the G1 phase [56]. PRKCE is involved in lipopolysaccharide (LPS)-mediated signaling in activating macrophages and also functions in controlling anxiety-like behavior [57]. The protein product of CSF3 is cytokine that controls production, differentiation and functions of granulocytes [58]. Therefore, molecular interactions of P38 MAPK with T-cell receptor (TCR), B-Cell Receptor (BCR) complex, and interferon gamma may be impaired due to deletion of CDKN1A, PRKCE, CSF3 and other CNV related genes. It might indicate for suppression of cellular response leading to reduced heterophil counts in LS birds of quail.

We identified different sets of genes affected by CNVs in HS and LS lines of quail, most importantly involved in nervous and endocrine systems development, humoral and cell-mediated immune response and different metabolisms. This result supports our hypothesis that CNVs have impact in increasing genotypic diversity and thereby phenotypic traits observed in quail. In the future we will perform a functional validation study such as expression of candidate CNV genes at protein level using different tissues from the two quail lines. The quail will continue to evolve as an important research animal model for understanding well-being and production performances in avian species and other animals.

## Supporting information

S1 Table Lists of Copy number variation regions (CNVRs) in HS and LS lines of Japanese quail. (XLSX)

S2 Table Lists of uniquely deleted and duplicated genes in HS and LS lines of Japanese quail. (XLSX)

S3 Table List of high copy number between HS and LS lines of Japanese quail. (XLSX)

S4 Table Gene networks associated with CNVs in HS and LS lines of Japanese quail. (XLSX)
